# Fabrication and Quantification of Chromium Species by Chemical Simulations and Spectroscopic Analysis

**DOI:** 10.3390/molecules31030506

**Published:** 2026-02-02

**Authors:** Abesach M. Motlatle, Tumelo M. Mogashane, Mopeli Khama, Tebatso Mashilane, Ramasehle Z. Moswane, Lebohang V. Mokoena, James Tshilongo

**Affiliations:** 1 Analytical Chemistry Division, Mintek, Private Bag X3015, Randburg, Johannesburg 2125, South Africa; tumelom@mintek.co.za (T.M.M.); tebatsom@mintek.co.za (T.M.); ramasehlem@mintek.co.za (R.Z.M.); lebohangm@mintek.co.za (L.V.M.); jamest@mintek.co.za (J.T.); 2 Mintek Pyrometallurgy Division, Randburg, Johannesburg 2125, South Africa; mopelik@mintek.co.za

**Keywords:** chromium speciation, emission spectroscopy, chemical simulation, environmental monitoring, analytical modelling

## Abstract

Chromium (Cr) exists in multiple oxidation states, with Cr(III) and Cr(VI) being the most environmentally and industrially relevant due to their distinct toxicity profiles and chemical behaviour. This study presents a comprehensive method that combines chemical simulation modelling, emission spectroscopy for quantification, and the controlled laboratory production of Cr species. Key findings include that acid digestion effectively extracted the Cr(III) and total Cr species, while thermodynamic modelling forecasted their stability and speciation under various environmental conditions. Thematic analysis indicates that the current quantification of Cr species is still in early development and remains centralized. Mineralogical and surface investigations showed that samples 1 and 2 have a BET surface area below 1 m^2^/g, whereas samples 3 and 4 exceed this. All samples are crystalline, with approximately 54.3 weight percent Cr_2_O_3_, 7.3 weight percent SiO_2_, 17.75 weight percent of MgO, and 8.3 weight percent Al_2_O_3_, suggesting Al and Fe^2+^ replacement of Cr in the spinel structure. Computational fluid dynamics (CFD) modelling revealed that longer residence times are necessary for higher Cr metallization under H_2_-CH_4_-reducing conditions, and accurately predicted carbon deposition on pellets. These results demonstrate that CFD can optimize the H_2_:CH_4_ ratio to minimize carbon deposition and enhance gas transport to reaction sites.

## 1. Introduction

Chromium (Cr) is a transition metal of significant environmental, biological, and industrial importance [1,2]. Trivalent chromium [Cr(III)] and hexavalent chromium [Cr(VI)] are the most prevalent and environmentally significant of its several oxidation states. While Cr(VI) is extremely poisonous, mutagenic, and carcinogenic, Cr(III) is a necessary micronutrient in trace amounts [3,4]. Analytical techniques that maintain natural chemical forms of chromium during sampling, preparation, and analysis are necessary for accurate chromium speciation. The lack of capacity of conventional methods to distinguish between Cr(III) and Cr(VI) in total chromium measurements could result in totals that are inaccurate [5]. Thus, method development is required to guarantee species stability, sensitivity, and selectivity [6,7]. It is crucial to fabricate or synthesize chromium species in a controlled laboratory setting to examine their behaviour and interactions in intricate systems [8]. Cr(III) species can be produced selectively using well-defined chemical processes to act as standards or to mimic actual situations, including contaminated soils, geological samples, and rivers or industrial effluents [9,10,11].

Although species interconversion and matrix interferences make it difficult to quantify chromium species at low levels in complex matrices, new developments present new analytical possibilities [12,13]. Cr has been determined using a variety of approaches, from sophisticated spectroscopic procedures to colorimetric and electrochemical methods [14]. Due to its great sensitivity, accuracy, and capacity to work with complicated matrices, emission spectroscopy, and particularly inductively coupled plasma–optical emission spectroscopy (ICP-OES) has become a reliable technique, and is integrated into chromium speciation to allow for reliable quantification, fast analysis, and simultaneous multi-element detection in both solid and aqueous samples [15,16,17]. By offering details on oxidation states and coordination environments, methods including X-ray diffraction spectroscopy (XRD) supplement emission techniques [18]. These revelations are essential to comprehend the interactions of Cr with biological systems, sorbents, and ligands. Spectroscopic signals improve the accuracy of speciation analyses by aiding in the differentiation of Cr(III), especially in heterogeneous or multi-phase systems [19].

Chemical modelling and simulation tools, such as thermodynamic equilibrium software (FactSage 8.3) and density functional theory (DFT), have become indispensable in modern analytical chemistry [20]. Through the use of transmission electron microscopy (TEM) and Brunauer–Emmett–Teller (BET) analytical techniques, scientists can forecast how Cr species will behave in a range of experimental and environmental settings [21,22]. By determining stable species, forecasting reaction paths, and assessing possible interferences, modelling aids in experimental design. Simulation improves the interpretation of analytical data and offers a theoretical framework for comprehending transformation mechanisms and speciation dynamics in the context of Cr speciation [20]. Cr species are widely studied due to their applications in diverse fields [23,24]. Furthermore, Cr speciation plays a key role in waste treatment, occupational health research, and environmental monitoring [25,26,27,28,29]. The chemical industry applies to metal in a variety of chemicals, such as catalysts and intermediates, which are produced using Cr compounds [30]. In the electronics industry, Cr’s use is limited by its conductivity; however, it is still employed in the manufacturing of electrical contacts and various electronic components [31,32].

This study was aimed at establishing a comprehensive methodology for the fabrication and quantification of Cr species by integrating chemical simulations with emission spectroscopy and surface morphology interpretation. Predictive chemical modelling tools are used to assess the stability and interactions of pure Cr(III), which are synthesized in a controlled laboratory environment. Even in intricate industrial and environmental matrices, emission spectroscopy’s accurate quantification makes it possible to investigate Cr species in extensive detail. BET surface area and surface morphology are complementary metrics. BET surface area measures the accessible surface that contributes to adsorption and reactivity, whereas surface morphology offers structural and visual insight into surface features. BET surface area frequently changes in tandem with morphological analysis-observed changes in particle size, porosity, or surface roughness. Through the integration of modern spectroscopic techniques, simulation, and experimental fabrication, this study improves the accuracy, dependability, and generalizability of chromium speciation analysis in a variety of applications.

### Modelling the Pre-Reduction of Chromite Pellets

Pre-reduction of chromite pellets with a mixture of H_2_ and CH_4_ is characterized by several phenomena, such as kinetic and mass transfer limitations and the deposition of carbon on the reaction sites, which blocks access to reducing gases. To optimize the process, an in-depth study of the governing phenomenon is required. Computational fluid dynamics (CFD) enables studies that aim to relate chemical kinetics, transport phenomena, reactor configuration, flow field, particle size, porosity, and degree of reduction. With CFD models, it is possible to determine the reducing gases flow rate that overcomes the mass transfer limitations; the optimum ratio of CH_4_:H_2,_; the optimum particle size; and operating temperature.

The chromite pellets were considered spherical, and the flow through and within the particle was resolved. The governing equations that describe momentum, continuity, species conservation, and energy balance for the gas phase are presented in Equations (1)–(4). The gas–solid reactions are represented by the source term R.

(1) $$\frac{\partial }{\partial t}\rho^{G}u\;+\;\nabla .\left(\rho^{G}uu\right)+\varepsilon \nabla P-\nabla .\left(\mu_{eff}\nabla u\right)-\rho^{G}g=-\mu_{eff}D.u-F.u$$

(2) $$\frac{\partial }{\partial t}\varepsilon \rho^{G}+\nabla .\left(\rho^{G}u\right)=\left(1-\varepsilon \right)\sum_{i}R_{i}^{Reduction}$$

(3) $$\frac{\partial }{\partial t}\varepsilon \rho^{G}y^{G}+\nabla \left(\rho^{G}uy^{G}\right)-\nabla .\left(\varepsilon \rho^{G}D_{eff}\nabla Y^{G}\right)={\varepsilon w}^{G}+\left(1-\varepsilon \right)R_{i}^{Reduction}$$

(4) $$\frac{\partial \left(\varepsilon \rho^{G}{Cp}^{G}T^{G}\right)}{\partial t}+\nabla .\left(\rho^{G}{Cp}^{G}T^{G}u\right)-\nabla .\nabla \left(\varepsilon k_{eff}^{G}T^{G}\right)=-\varepsilon \sum_{i}w_{i}h_{f,k}^{0}-h_{conv}S_{Av}\left(T^{G}-T^{S}\right)\;\;\;\;\;\;\;\;\;\;\;\;\;\;\;\;\;\;\;\;\;\;\;\;\;\;\;\;\;\;\;\;\;\;\;\;\;\;\;\;\;\;\;\;\;\;\;\;\;\;\;\;\;\;\;\;\;\;\;\;\;\;\;\;\;\;\;\;\;\;\;+\left(1-\varepsilon \right)T^{G}\sum_{i}{Cp}_{i\;}R_{i}^{Reduction}+S^{G,Radiation}$$

In Equations (1)–(4), Cp is the specific heat, k_eff_ is the gas thermal conductivity, D denotes Darcy’s resistance to flow, G is the gas phase, ε is porosity, D_eff_ is the effective diffusion coefficient, ρ denotes density, u is the gas velocity, and μ_eff_ is the dynamic viscosity. The subscript eff denotes the contributions of correlations resulting from volume averaging.

The solid-phase governing equations are presented in Equations (5)–(7), and in this case, the momentum equation is not considered since the study considered a fixed bed of chromite particles. The interaction between the gas and solid phase was imposed through a reaction rate source term (R).

(5) $$\frac{\partial }{\partial t}\left(1-\varepsilon \right)\rho^{S}=\left(1-\varepsilon \right)\sum_{k}R_{K}^{Reduction}$$

(6) $$\frac{\partial }{\partial t}\left(1-\varepsilon \right)Y_{k}^{s}\rho^{S}=\left(1-\varepsilon \right)R_{K}^{Reduction}$$

(7) $$\frac{\partial }{\partial t}\left(1-\varepsilon \right)\left(\rho^{S}C_{p}^{s}T^{S}\right)-𝛻.\left(\left(1-\varepsilon \right)Kk_{eff}^{s}.𝛻T^{S}\right)=h_{conv}S_{Av}\left(T^{G}-T^{S}\right)+Hr+S^{S,Radiation}$$

where $R_{k}^{Reduction}$ denotes the reduction rate of the solid species and $R_{i}^{Reduction}$ denotes the reduction rate of gas species.

He, S. et al. [33] describes a porous medium as a collection of scalar and tensor properties and developed a standalone CFD solver that couples chemistry with transport phenomena and describes the structural changes in pellets as pre-reduction progresses. Their approach was used in the current study to describe the pre-reduction of chromite pellets with a mixture of CH_4_ and H_2_.

## 2. Results

### 2.1. Quality Control

The highest level of precision and accuracy was ensured by the laboratory’s ISO/IEC 17025 accreditation [34], which was used for all the experiments and analyses. This accreditation guarantees that the tools and processes adhere to stringent quality control guidelines to yield precise and consistent outcomes. Check samples confirmed the dependability of the data while also confirming that there were no matrix effects or instrument drift. Consistent instrument performance was ensured during the run by routinely inspecting quality control samples. Several standard solutions with known concentrations were used to build the ICP-OES calibration curve.

### 2.2. Bibliometric Analysis

**Figure 1:**
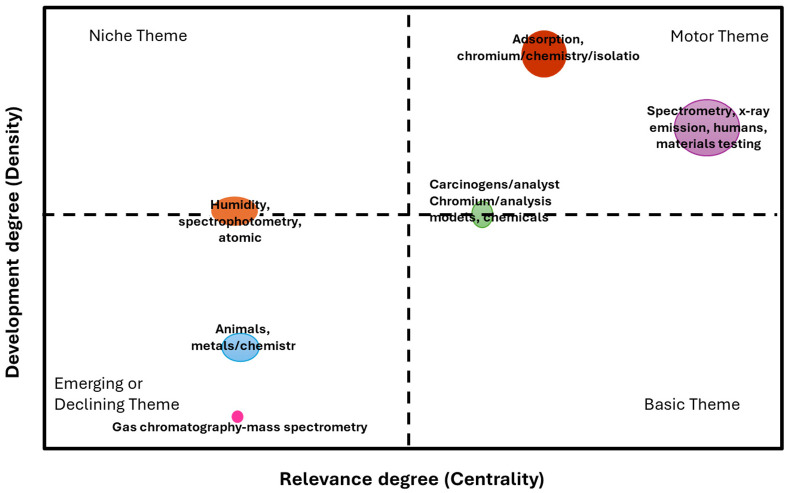
The thematic map depicting the relevance degree and research and development progress from the year 2000 to 2025.

Thematic maps display spatial distributions (Figure 1), which are frequently demographic, social, cultural, or economic, and are represented based on the topic. The development and relevance degree are the most well-known types of thematic maps, where regions are coloured based on how a data variable is distributed. The different themes presented measure the density vs. the centrality of the chromium speciation, current applications, and gaps. It is challenging to identify abnormalities, comprehend the underlying events, and derive useful knowledge without looking at the data. Wu et al. [35] reported on the research progress of heavy metals in the desert via a visual analysis based on Cite Space. The researchers found that since its debut in 2000, the field’s literature has continuously grown in quantity. Most of the literature was published in reputable journals, the United States led the world in the quantity of papers published, and the findings of research from industrialized nations had a bigger impact. With the greatest centrality and the greatest number of published articles, Chinese Academic Science has made exceptional contributions to the field and is currently at the forefront of it. The reality is that research institutions do not cooperate or communicate with one another. Furthermore, acquiring knowledge about these challenges and disseminating it to the public is facilitated by the visual examination and presentation of the underlying spatial data.

### 2.3. Modelling

**Figure 2:**
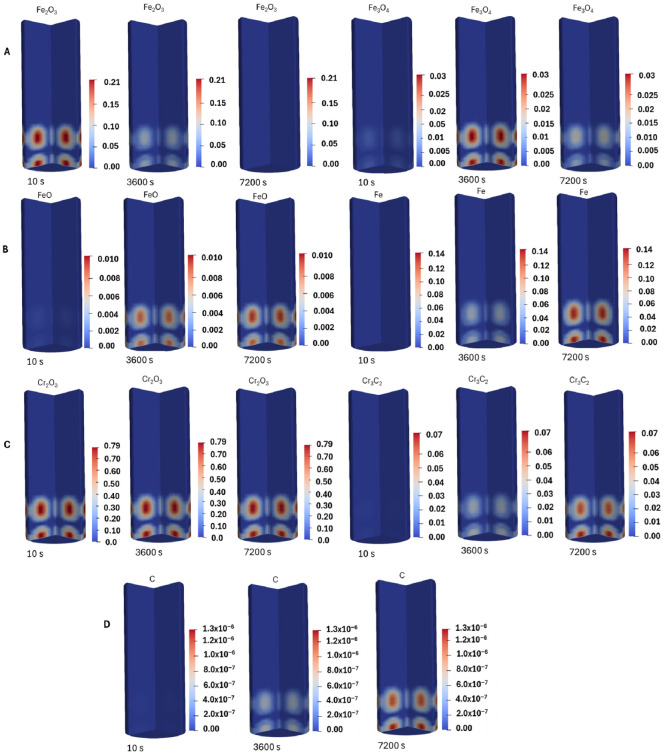
(**A**) Fe_2_O_3_ and Fe_3_O_4_ mass fraction profiles, (**B**) FeO and Fe mass fraction profiles, (**C**) Cr_2_O_3_ and Cr_3_C_2_ mass fraction profiles, and (**D**) carbon mass fraction profiles.

The mass fraction profiles of species are shown in Figure 2. The results shown are for the isothermal pre-reduction of chromite pellets at 1000 °C with a mixture of H_2_ and CH_4_. The ratio of partial pressure of CH_4_:H_2_ was 25:75, and this was chosen to eliminate excess carbon deposition on the reaction sites. The findings show that after 2 h of pre-reduction at 1000 °C, not all iron oxide and chromium oxides were reduced, indicating that longer reduction times are required. This is evident from the mass fraction profiles. In their study, Xu et al. [36] demonstrated that the pre-reduction of chromite under CH_4_ begins with the adsorption of CH_4_ on the active sites of the oxide, followed by its decomposition into carbon and H_2_. They further stated that, under appropriate CH_4_:H_2_ mass ratios, the activity of carbon from CH_4_ decomposition is above unity. This creates favourable conditions for the reduction to carbides to occur at lower temperatures (925 °C), and this is evident in Figure 2D, where Cr_3_C_2_ is formed from the pre-reduction reactions. The study by Anacleto et al. [37] revealed that under CH_4_-reducing environment, the correlation between the rate of metallization and temperature is not significant; instead, there is a strong correlation between the rate of metallization and CH_4_ partial pressure.

At the chosen CH_4_:H_2_ ratio in the current study, carbon deposition in the reaction site is minimal, and these results demonstrate the capability of CFD models in predicting the optimum operating conditions for maximizing the degree of reduction. The results presented demonstrate how crucial it is to integrate computational and analytical methods for increased accuracy in trace metal analysis using Equations (1)–(7). In order to account for the inhomogeneity of the porous media and the porosity of the pre-reduced product predicted by the CFD model, a non-uniform porosity distribution will be considered in the future. Currently, the models assume a homogeneous porous medium. The introduction of Darcy’s term into the Navier–Stokes equation to account for the pressure drop in the porous medium is demonstrated in Equation (7). Equations (4) and (5) show the governing continuity and momentum equations, whereas the Eulerian framework characterizes the gas phase. The heat source term and the source term (R) ensure the coupling between the gas and solid phases.

The models currently assume a homogeneous porous medium, but in the future, a non-uniform porosity distribution will be taken into account to reflect the inhomogeneity of the porous medium, and the porosity of the prereduced product predicted by the CFD model will be validated using a micro-CT-based approach.

### 2.4. Structural and Morphological Characterization

#### 2.4.1. BET

The analysis of nitrogen adsorption and desorption was performed to assess the samples’ specific surface area. When assessing the chromate ore’s filtering performance and structural characteristics, the BET (Brunauer–Emmett–Teller) study offers valuable information on its surface area, pore size, and pore volume. Table 1 lists the BET analysis parameters for samples 1, 2, 3, and 4. Because of its structure, chromate is known to have a relatively low specific surface area of less than 1.0 m^2^/g according to the BET analysis. Depending on the chemical analysis, the pore size distribution frequently varies between 30 and 100 Å, suggesting a mesoporous-to-macroporous nature appropriate for applications involving microfiltration. The BET measurements demonstrate a notable constant value in surface area with a pore volume of less than 0.1 for all the samples. Wu et al. [38] investigated the impact of physical structure on the toxicity of chromium (VI) residues and revealed that the adsorption of the chromium (VI) compound at the residue’s surface is the primary source of the toxicity, and that the specific surface area determines the chromium concentration. Since materials with larger surface areas offer more reactive sites for dissolution, adsorption, desorption, and redox reactions, the BET surface area is a crucial factor affecting chromium leachability and environmental risk. However, the BET surface area alone cannot be used to evaluate the environmental effect. Because Cr(VI) is far more poisonous and mobile than Cr(III), chromium speciation is important. The link between BET surface area and chromium release is still unclear in the absence of clear speciation data and leaching investigations. Therefore, the combined assessment of surface area, chromium speciation, and leachability under environmentally relevant conditions is necessary for meaningful environmental risk assessment.

#### 2.4.2. X-Ray Diffraction Analysis

**Figure 3:**
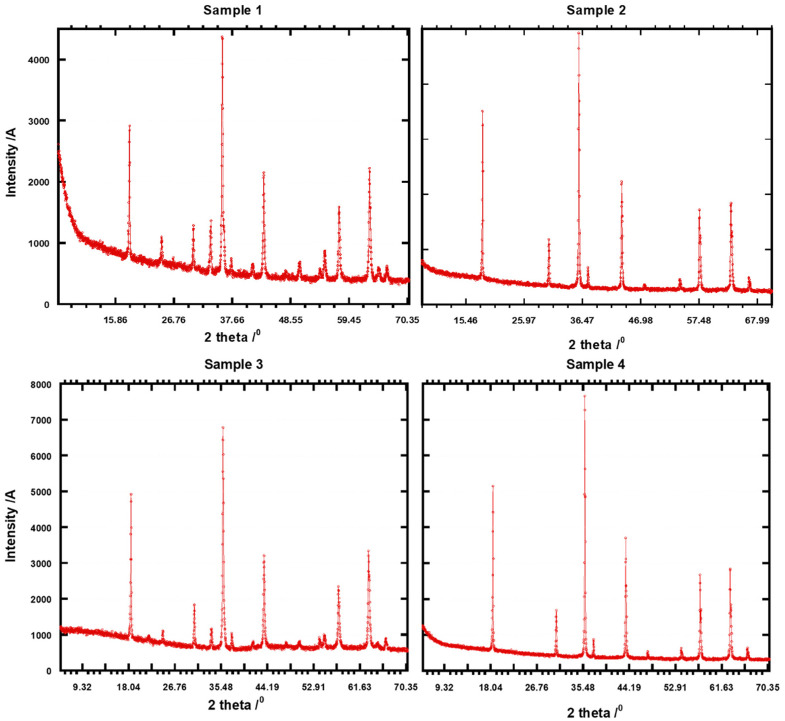
XRD spectra showing the different mineral phases of samples 1, 2, 3, and 4 of chromate ore processed for Cr speciation.

The mineral phases in the various size fractions determined by XRD are shown in Figure 3. The chromate ore is distinguished from natural soil by a prominent calcite peak that is extremely abundant in quartz [39]. The mineralogical analysis showed that the samples are crystalline; this is seen from the prominent peaks for samples 1, 2, 3, and 4, with no amorphous phase observed. However, the precise peak positions and intensities can vary depending on the specific composition of the chromite ore, including the presence of impurities like silicate minerals (e.g., enstatite, plagioclase), which may show additional peaks on the XRD pattern [40]. The dominant peaks in chromite ore correspond to the spinel structure of chromite (FeCr_2_O_4_), with the most prominent peaks typically observed around 2θ values of 35.5°, 57.0°, and 62.5°. Regarding chromate minerals, the greater observable peak strengths for periclase, chromite, and brownmillerite in the coarse sand-sized fractions are indicative of the chromate parent materials [41]. Hsu et al. [42] reported a study of the synchrotron X-ray analysis of chromium speciation in the size fractions of a soil contaminated by weathered chromate ore process residue. The findings showed that Cr^3+^ dominated three size fractions and was present in both COPR parent minerals and hydrated derivatives. The synchrotron data showed organic-matter-bound Cr^3+^, particle-adsorbed Cr^3+^, and chromite-dominated Cr^3+^.

#### 2.4.3. Transmission Electron Microscopy

**Figure 4:**
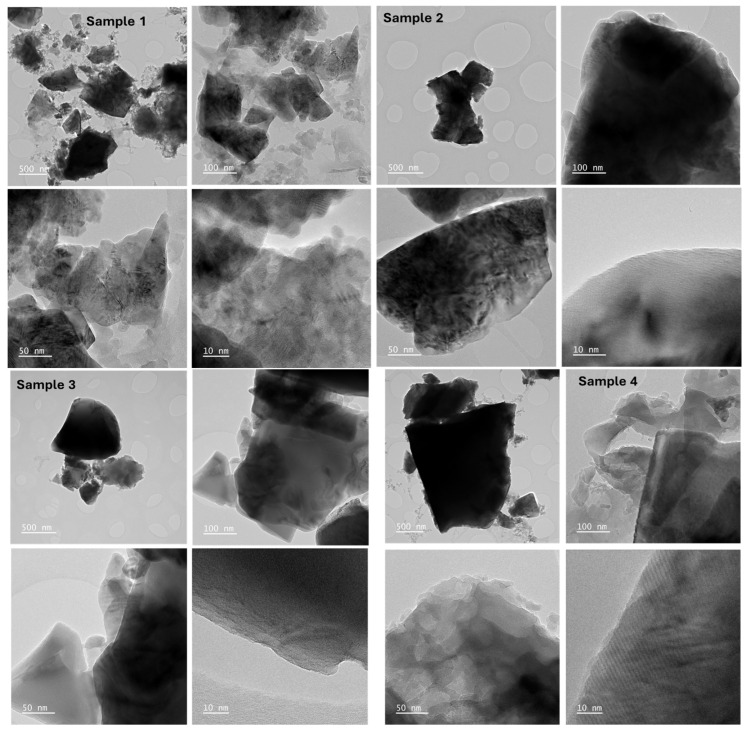
TEM micrographs for samples 1, 2, 3, and 4 at magnifications of 500, 100, 50, and 10 nm.

The morphology and structure of chromate precipitates produced during the reduction of chromate ore to Cr and Fe species can be described using TEM. Figure 4 displays the morphologies of chromate ore after processing, which is attributed to the absorption capacity of electrons differently than organic and inorganic components. The structure showed spherical dense dots connected on both the surface and background for sample 1 and sample 4, with bright areas signifying the impurities. Regular pentagon shapes are observed, are mono-dispersed (i.e., very uniform in size), and seem to be well-proportioned. Sample 2 shows a more crystalline structure, with brick-like morphology and a less amorphous phase. Sample 3, on the other hand, shows an equal part of amorphous and crystalline structure with a square-like structure. Nonetheless, certain particles tend to group due to their high surface energy, frequent collisions [43], and interactions between dipoles. The crystallographic analysis determined by the XRD and the TEM morphology agrees quite well. Ahmed et al. [44] assessed the carbothermic reduction of chromite ore via thermal analysis using a mill scale. The researchers found that the inclusion of mill scale in the alloying mixture enhances the reduction of chromite ore, particularly at temperatures over 1623 K. The mill scale is first reduced to high active iron and then distributed around the chromite ore particles. This effect is ascribed to the presence of a molten Fe–C alloy near chromite ore, which can cause chromium to dissolve in situ into the melt and reduce its thermodynamic activity.

### 2.5. Spectroscopic Analysis

#### ICP-OES

Table 2 below shows the ICP-OES metal oxide concentrations for samples 1, 2, 3, and 4. To account for reproducibility and quality control, the findings are shown in duplicates. Samples 1, 3, and 4 are similar in the assay, and sample 2 shows a different composition. According to surface chemical analysis, the ore included 7.3 weight percent SiO_2_ and approximately 54.3 weight percent Cr_2_O_3_ (Table 2). Significant amounts of Al_2_O_3_ (8.3 weight percent) and MgO (17.75 weight percent) were also found in the sample, indicating that Al and Fe^2+^ have significantly replaced Cr in the spinel structure. Calcium (0.63 weight percent), manganese (0.15 weight percent), and titanium (0.15 weight percent) were among the minor contaminants. Manganese, titanium, and vanadium can all have limited solid substitution in the spinel structure, while calcium and sodium are most likely linked to gangue mineral grains like feldspars and pyroxenes. To identify any size-based variations in bulk chemistry, ICP-OES was used to investigate the assay data. Sample 2 is high in CaO, SiO_2_, and Al_2_O_3_ with 42.2, 22.45, and 18.4 weight percent, respectively, and MgO with 7.33 weight percent. The Cr and Fe are significantly low, attributed to the high clay content. The methods of analysis used have a significant impact on the Cr(III) concentrations shown in Table 2. Measurable differences in the measured Cr(III) levels were caused by variations in sample preparation, extraction conditions, and analysis procedures. While softer techniques mirrored the more easily accessible Cr(III) fraction, more aggressive extraction conditions tended to produce larger Cr(III) concentrations, indicating increased mobilization of chromium from the solid matrix. These differences show how effective each experimental technique is at focusing on particular chromium pools, proving that the method choice is crucial for interpreting Cr(III) speciation findings. As a result, the comparative analysis of the techniques shown in Table 2 sheds light on how well they work for determining Cr(III) under various analytical goals.

### 2.6. Quantitative Speciation and Simulation Comparison

#### Correlation Between CFD Predictions and Measured Speciation

**Figure 5:**
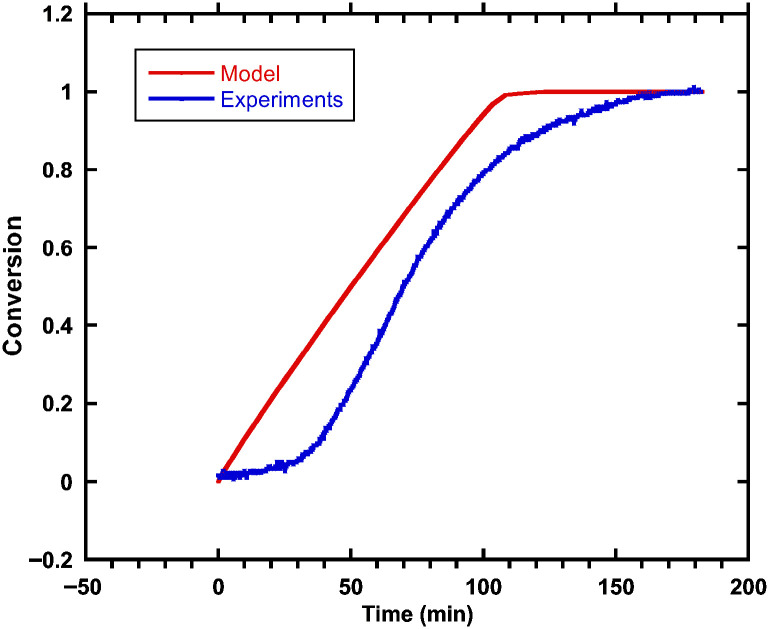
Comparison between experimental and model results.

A comparison between experimentally detected species and predictions derived from CFD simulations was carried out to verify the speciation behaviour of chromium during the pre-reduction of chromite pellets using a H_2_/CH_4_ gas mixture. To replicate the dynamic reduction environment, the CFD model combined reaction kinetics, heat and mass transfer, and gas–solid interactions inside the pellet matrix. Comparison between the conversion calculated from experimental measurements and predicted from the CFD model is presented in Figure 5. As observed, there are some differences in the conversion profiles, and this could be attributed to the model not capturing the transport limitations accurately, as evidenced by the faster conversion from the model when compared to experimental measurements.

ICP-OES measurements for chromium species were used to confirm the model’s correct predictions of the transition from chromate ore to Cr(III) under various gas compositions and temperatures. The comparison showed that the experimental and anticipated chromium speciation were highly correlated, especially at reduction temperatures between 1000 and 1200 °C. The function of H_2_ as a more efficient reductant than CH_4_ under the study conditions was confirmed by the model’s prediction of enhanced Cr(III) production with higher H_2_ concentrations, which matched spectroscopic results. However, slight variations were noted in atmospheres rich in CH_4_, most likely because of kinetic constraints and incomplete accounting for carbon deposition in the equilibrium-based model. According to these findings, CFD simulations provide a reliable predictive framework for speciation investigations in high-temperature chromite processing settings when calibrated with experimental data.

## 3. Materials and Methods

### 3.1. Reagents

All reagents were of analytical grade and were utilized without further processing. The reagents were all obtained from Associated Chemical Enterprises Chemicals Pty Ltd. (Johannesburg, South Africa, 2091). Anhydrous methanol and 5% bromine–methanol solution were used for the preparation of metallic iron (Fe). A sulphuric–phosphoric acid mixture and hydrochloric acid, 32–37%, respectively, were used for the preparation of metallic Cr.

### 3.2. Characterization and Bibliometric Analysis

Data analysis was performed using bibliometric RStudio (version 4.4.2), which is a technique for evaluating publications using statistical techniques to comprehend the significance and impact of academic work. Bibliometric analysis evaluates the production and impact of the research; it entails counting the number of publications, citations, and other metrics. As a result, chemical reactions are quantitatively represented, and simulation was employed as a method to understand and predict their behaviour. To monitor the concentration of species over time, including other macro- and micro-properties, this entails establishing reaction mechanisms and solving the governing equations, which include momentum, continuity, species conservation, and energy equations. Modelling aids in understanding the effects of various parameters on process performance, predicting reaction rates, and determining ideal settings.

The BET (Brunauer–Emmett–Teller) method was used to measure the quantity of gas adsorbed on the surface of solid and powder samples to calculate their specific surface area. Surface area and pore size of the CCMs were determined by the BET method. A Micrometrics TRISTAR 3000 surface-area analyser (Malvern Panalytical, Jan Frederick Ave, Randburg, Johannesburg, South Africa) was set up using the low-temperature N_2_-adsorption method. Before analysis, each sample was degassed at 100 °C for 4 h under a continuous flow of N_2_ gas to remove volatile moisture and adsorbed contaminants.

X-ray diffraction (XRD) was used for examining the phase composition, crystal structure, and orientation of powder and solid samples. The structural analysis of the dry films was characterized using a PANalytical XPERT-PRO diffractometer using Ni-filtered CuKα radiation (λ = 1.5406 Ǻ) with a fixed slit at 45 kV (voltage) and 40 Ma (current) in the 2θ diffraction angle range of 4.5 to 90° (Malvern Panalytical, Jan Frederick Ave, Randburg, South Africa).

A transmission electron microscope (TEM) is a powerful microscopy device that produces a substantially magnified image by passing an electron beam through a tiny material, exposing details at the microscopic level. The species was characterized using a Jeol model 2100 TEM with a 200 kv acceleration voltage and a LaB6 filament in the bright field mode (JEOL USA, Dearborn Road, Peabody, MA, USA). The carbon powder samples were dispersed in ethanol and sonicated for 5 s before dipping the TEM carbon grid in the solution and allowing it to dry in air before the analysis.

Metal oxides and elemental concentrations were measured using an Agilent 5900 Series ICP-OES spectrometer (Agilent Technologies, Santa Clara, CA, USA). The parameters of the ideal settings for ICP-OES are as follows: RF power—1250 W, nebulizer gas flow—0.7 L/min, auxiliary and plasma gas flow—1.2 L/min, integration time—1 and 6 s, pump speed—10–12 mL/min, sample uptake—1.0–1.6 mL/min, rinse time—40 s, and internal standard—scandium. The standards and sample dilutions were made using ultra-pure de-ionized water with a resistivity of 18 MΩ-cm, which was produced by the Milli-Q Direct 16 purification system (Darmstadt, Germany).

### 3.3. Experimental

The samples, which range in temperature from 1000, 1200, 1400, to 1500 °C, were labelled as samples 1, 2, 3, and 4. The ore samples were reduced prior to extraction in order to assess the impact of temperature.

#### 3.3.1. Extraction and Fabrication of Chromium and Iron Species

##### Iron Species

Analysis of Fe species was conducted following the wet chemistry laboratory method that describes the leaching of metallic iron. The sample was treated with a bromine–methanol solution for the selective dissolution of metallic iron in the presence of its oxides. Approximately 0.5 g of the dry sample was weighed into an empty 250 mL conical beaker. A total of 50 mL of brominated methanol solution was added from a dry measuring cylinder. The beaker was covered with a watch glass, and the solution was continuously stirred for 1 h using a magnetic stirrer at room temperature. The watch glass was rinsed with methanol and filtered immediately through a double glass-fibre filter under suction, and the filtrate was collected in a Buchner flask. The filter was then washed with methanol until the washings were free of bromines. The filtrate was transferred to a 500 mL conical beaker and the flask was rinsed well with water. A total of 20 mL of sulphuric acid (50%) and a few anti-bumping granules were added, and the solution evaporated until there were less fumes. The beaker was removed from the hot plate, cooled, and 10 mL of hydrogen peroxide was added, and the solution was evaporated until dense white fumes evolved. The solution was then cooled and a further 10 mL of hydrogen peroxide solution was added. The watch glass and the walls of the beaker were rinsed with deionized water and finally evaporated to fumes. A total of 100 mL of water and 20 mL of concentrated hydrochloric acid were carefully added and boiled until all the salts had dissolved. Then, 5 mL aliquot was transferred into a 100 mL volumetric flask; 10 mL of concentrated hydrochloric acid was added and diluted to the mark with deionized water.

##### Chromium Species

The primary method for distinguishing different chemical forms of chromium is speciation analysis, which often combines advanced analytical techniques with selective chemical reactions to discriminate between Cr(VI) and Cr(III). Using a separation technique called selective extraction, the Cr(III) and Cr(VI) species are first physically separated. The separated species are then placed in a very sensitive detector, typically the Agilent (Agilent technologies) 5900 inductively coupled plasma–optical emission spectrometer (ICP-OES), Penang, Malaysia or an inductively coupled plasma–mass spectrometer (ICP-MS) (iCAP MX SERIES), Waltham MA, USA. To distinguish the different chemical forms (species) following separation/extraction, we allowed for the quantification of each species independently before detection. The determination of Cr species was conducted following the developed wet chemistry method that describes the chromium and iron metallic phase being selectively leached by digestion with a mixture of sulphuric and phosphoric acids. The method applies to semi-reduced chromite products, with variable content in metallic iron and metallic chromium. Approximately 0.5 g of the sample was accurately weighed and transferred to a 250 mL polytetrafluoroethylene (PTFE) beaker, and 50 mL of sulphuric–phosphoric acid mixture was added to the sample. The solution was heated on a hot plate for 1 h and filtered under suction through double glass fibre filter papers into a large Buchner funnel and washed well with water. The filtrate was transferred to a 200 mL flask, diluted to volume with water, and mixed. A 5 mL aliquot was transferred into a 100 mL volumetric flask; 10 mL of concentrated hydrochloric acid was added and diluted to the mark with deionized water.

##### Peroxide Fusion for Metal Oxides Analysis

About 0.2 g of the sample was combined with 1.5 g of sodium peroxide and 0.5 g of sodium carbonate in a zirconium crucible for peroxide fusion. Using a Bunsen burner, the mixture was fused until it melted completely. After cooling, the samples were dissolved in 100 mL deionized water inside a 250 mL beaker. A total of 40 mL of concentrated HCl was added to the beaker to leach the sample. A plastic rod was used to remove the crucible from the beaker, and it was carefully rinsed and cleaned with deionized water. The solution was transferred into a 200 mL volumetric flask containing 10 mL of 0.2 g/L scandium internal standard. To accurately determine the amounts of metal oxides, the sample was subjected to ICP-OES analysis.

#### 3.3.2. Calibration

Multi-element standard solutions were used for ICP-OES calibration, and quality control solution analysis was used to validate the calibration curves that were produced. With the correlation coefficients (R^2^ > 0.999) employed across the concentration range, the ICP-OES technique showed outstanding linearity. The development of linear calibration curves for ICP-OES over a broad concentration range (0.005–0.2%), especially in samples with higher analyte concentrations, allowed for accurate measurement.

#### 3.3.3. LOD and LOQ

ICP-OES was used to analyze blank samples and steadily decreasing amounts of standards to determine the limit of detection (LOD) and limit of quantitation (LOQ) for metal oxides and Cr determination. Following Equations (8) and (9), the LOQ was determined to be three times the LOD, and the LOD was determined to be three times the standard deviation of the blank signal (*n* = 10). The sensitivity and accuracy of metal oxide detection were ensured by optimizing these parameters to allow precise readings at trace levels.

LOD = 3 × SDBLANK(8)

LOQ = 3 × LOD(9)

#### 3.3.4. Quality Control/Quality Assurance (QC/QA)

The implementation of a QA/QC programme was necessary to guarantee the production of reliable results. The QA/QC process involved performing the experiments in triplicate, and the mean value of the outcomes was reported. The data was considered acceptable when the percentage difference and percent error within the triplicate samples were less than 10%.

### 3.4. Modelling

The governing equations describing pre-reduction of chromite pellets with a mixture of CH_4_ and H_2_ were solved numerically using a standalone CFD solver for reacting flows. The simulations were conducted using the OpenFOAM^®^ v2406 computational fluid dynamics (CFD) code. The geometry and mesh were input, including the depiction of how pellets were embedded into the geometry. In order to facilitate the reduction in the mass transfer to the pellets’ reaction sites, pellets were placed with some space in between.

## 4. Conclusions

This study effectively illustrated the fabrication of chromium species and their quantification using emission spectroscopy and chemical simulation methods. This method made it possible to distinguish between Cr (III) with accuracy, and simulations confirmed the experimentally observed kinetic and thermodynamic behaviour. The ability of emission spectroscopy to quantify Cr species in a variety of matrices demonstrated its promise for industrial process control and environmental monitoring. These results highlight how crucial it is to combine analytical and computational techniques for improved trace metal analysis accuracy following Equations (1)–(7). The CFD model results indicate that longer residence times are required to achieve higher degrees of Cr metallization. Carbon deposition on the pellets was predicted by the CFD model, which is important since the model can predict the ideal H_2_:CH_4_ ratio that reduces carbon deposition and improves gas flow to the reaction sites.

Bibliometric analysis showed that the quantification of Cr species is still at the baseline of the development stage and is centralized. The BET surface area was less than 1 m^2^/g for samples 1 and 2, while samples 3 and 5 were greater than 1 m^2^/g. The mineralogical investigation revealed that the samples are crystalline. Surface chemical investigation revealed that the ore contained roughly 54.3 weight percent Cr_2_O_3_ and 7.3 weight percent SiO_2_. The sample also contained notable concentrations of MgO, 17.75 weight percent, and Al_2_O_3_, 8.3 weight percent, suggesting that Al and Fe^2+^ have substantially replaced Cr in the spinel structure. Computational fluid dynamics (CFD) was used to study pre-reduction behaviour of chromite under a reducing environment, and the model results indicate that longer residence times are required to achieve higher degrees of Cr metallization. The CFD model was able to predict carbon deposition on the pellets, which is crucial, as the model can predict the optimum ratio of H_2_:CH_4_ that minimizes carbon deposition and enhances the transport of gases to the reaction sites.

## Figures and Tables

**Table 1:** The BET results for the chromate samples.

SAMPLES	BET SURFACE AREA (m^2^/g)	PORE SIZE (Å)	PORE VOLUME (cm^3^/g)
Sample 1	0.9906	61.0873	0.0322 ± 0.0059
Sample 2	0.9061	39.2408	0.0452 ± 0.0079
Sample 3	1.6476	46.4335	0.0981 ± 0.0268
Sample 4	1.0482	57.2271	0.2710 ± 0.0570

**Table 2:** The mineral quantification of metal oxides by ICP-OES.

Sample	Al_2_O_3_ (%)	CaO (%)	Cr_2_O_3_ (%)	FeO (%)	MgO (%)	MnO (%)	SiO_2_ (%)	TiO_2_ (%)
1	8.31			12.6		0.16		
		0.63	54.3	12.6	17.9	0.16	7.28	0.15
	8.3	0.64	54.6		17.8		7.3	0.15
2	18.4			0.293				
	18.4	42.18	<0.07	0.305	7.32	6.18	22.6	
		42.27	<0.07		7.34	6.18	22.5	
3		1.05		12.7		0.15	6.65	0.17
	8.56	1.06	51.2	12.6	15.5	0.15		0.17
	8.54		51.1		15.5		6.82	
4	8.02		52	13.7	17.1	0.15	6.98	0.18

## Data Availability

Data available on request.

## Abbreviations

The following abbreviations are used in this manuscript:

Cr	Chromium
Fe	Iron
ICP-OES	Inductively coupled plasma–optical emission spectroscopy
XAS	X-ray absorption spectroscopy
UV-Vis	Ultra-violet spectroscopy
ISO/IEC	International Organization of Standards/International Electrotechnical Commission
BET	Brunauer–Emmert–Teller
XRD	X-ray refractive diffraction
FeCr_2_O_4_	Chromite
TEM	Transmission electron microscopy
PTFE	Tetrafluoreethylene
LOD	Limit of detection
LOQ	Limit of quantification
QA/QC	Quality assurance/quality control
CFD	Computational fluid dynamic
